# Does Male Care, Provided to Immature Individuals, Influence Immature Fitness in Rhesus Macaques?

**DOI:** 10.1371/journal.pone.0137841

**Published:** 2015-09-14

**Authors:** Doreen Langos, Lars Kulik, Angelina Ruiz-Lambides, Anja Widdig

**Affiliations:** 1 Junior Research Group of ‘Primate Kin Selection’, Department of Primatology, Max-Planck Institute for Evolutionary Anthropology, Deutscher Platz 6, 04103, Leipzig, Germany; 2 Institute of Biology, Faculty of Bioscience, Pharmacy and Psychology, University of Leipzig, Talstrasse 33, 04103, Leipzig, Germany; 3 Caribbean Primate Research Center, University of Puerto Rico, Medical Sciences Campus, PO Box 906, Punta Santiago, PR, 00741, United States of America; German Primate Centre, GERMANY

## Abstract

Among many mammals, maternal care strongly impacts infant survival; however, less is known about whether adult males also affect infant fitness. Paternal care is expected when providing care enhances offspring survival and reproduction, which likewise increases fathers’ fitness. Males might also care for unrelated immature individuals to increase their mating probability with the immature individuals’ mothers. Studies in multimale primate groups showed that sires enhance food access for offspring and provide protection in conflicts. Furthermore, fathers’ presence during infancy has been suggested to accelerate offspring sexual maturation. However, no study has yet directly linked the degree of father-offspring bonds to offspring fitness in primates. We previously reported father-offspring affiliation in rhesus macaques, pronounced during early infancy and independent of mothers’ presence. The present study aims at investigating whether affiliation with fathers or other males affects proxies of immature fitness (body mass gain, body fat and testis size). First, we combined behavioral, genetic and morphometric data from 55 subjects of one group. Second, using demographic and genetic data, we investigated for 92 individuals of the population whether mother- and father-offspring co-residence during immaturity influenced offspring lifetime reproductive success (LRS). Our results show that focal rank and higher amounts of affiliation with high-ranking males during infancy tend to positively impact body mass gain of female, but not male focal animals. In contrast, body mass gain of male focal individuals, but not females’, appeared to be higher when affiliation of male immature individuals was evenly distributed across their adult male partners. Moreover, we found mothers’, but not fathers’, presence during immaturity to predict offspring LRS. Our results suggest that male-immature affiliation, but not father-offspring co-residence, potentially impacts proxies of immature fitness. However, future studies should investigate the underlying mechanisms of male-immature relationships and their impact on immature fitness in more detail.

## Introduction

Among mammals, maternal effects (including maternal care) have been shown to influence the early life of offspring (reviewed in [1,2]). Even adults may benefit from their mother’s presence in terms of reproduction and survival (e.g., killer whales, *Orcinus orca*, [3], bonobos, *Pan paniscus*, [4]). Maternal effects in primates are particularly well studied, as maternal care is extended in duration compared to most other mammals [5,6]. The presence of mothers during early development [7–9] as well as maternal rank, age, behavior and sociality have been shown to be related to offspring condition (reviewed in e.g., [1]).

In contrast, the influence of paternal effects (including paternal care) on offspring fitness has been studied less, although it has been documented in several primate species [10–13]. Paternal effects are defined as the influence of fathers on the features of their offspring via mechanisms other than the transmission of genes [14]. These non-genetic paternal effects can be mediated by the transmission of epigenetic, somatic, morphological, behavioral, or environmental variants. Therefore, a paternal effect can occur when a non-genetic factor is transmitted from an adult male to its offspring, causing effects on offspring development [14]. Paternal care as the behavioral non-genetic paternal effect is defined as any care actively provided by adult males towards their young, which is supposed to be beneficial for the offspring. Hence, paternal care is expected to enhance survival and reproduction [15,16]. Paternal care can comprise close affiliation, infant carrying, protection and support during agonistic conflicts (reviewed in [15,17]).

Evidence for care provided by adult males mainly comes from monogamous species and those with one-male units (such as *Aotus* and *Callicebus spp*.), where paternity certainty is high. In these species, adult males (often the sire of an infant) predominantly contribute to infant care by carrying the young [15,17]. Paternal care has also been found in a species with a polyandrous mating system, the Geoffroy’s tamarin, *Saguinus geoffroyi* [18]. In this species, all males, which are closely related to each other (r = 0.36–0.44), mated with the female and later cooperatively cared for the infants, independent of sirehood. Male care in this species is mainly characterized by carrying young, but comprises any type of parental care behavior except lactation (e.g., affiliation, protection) [18]. Finally, paternal care has also been reported in multimale, multifemale groups, where females mate with multiple partners and paternity certainty is low, unless males mate-guard receptive females as a counter strategy for paternity confusion. For example, males affiliate more with their offspring than with unrelated infants (savanna baboons, *Papio cynocephalus* [19]; Assamese macaques, *Macaca assamensis* [20]; rhesus macaques, *M*. *mulatta* [21]) and preferentially support their offspring when involved in conflicts with other juveniles (savanna baboons [22]). Paternal care or the resulting male-offspring bonds have not yet been shown to enhance offspring fitness, except in a polygynous human population, where paternal investment correlated with offspring fitness [23]. However, several studies in non-human primates suggested paternal effects (other than paternal care) to impact offspring fitness. For example, in chacma baboons (*P*. *ursinus*), infants feeding in close spatial proximity to their sires acquired access to higher quality food patches than infants not feeding close to their sires [24]. Furthermore, a study in savanna baboons suggested an earlier onset of sexual maturation when the father was present in the group during the offspring’s immature period [25].

Selection is expected to favor paternal care when males can increase their own fitness by doing so [26,27]. However, infants should benefit from such care regardless of whether or not caretakers are the actual fathers. Indeed, in Barbary macaques (*M*. *sylvanus*) males are known to care for infants independent of their relatedness towards a given infant, either to increase their future mating success with the infants’ mothers (‘care-then-mate strategy’ [28]) or to regulate relationships amongst males [29]. In chacma baboons, male-infant relationships can be linked to both formation of friendships and paternity success [30,31]. Whereas male-female friendships in chacma baboons are often of short duration, mainly to ensure infant protection against infanticide [32–35], male-female bonds in Assamese macaques were found to be stable for several years [20]. Furthermore, mating success in Assamese macaques predicted friendship at parturition, but also male-infant association until weaning (ibid). However, the potential fitness benefits of paternal care remain unclear.

In this study we investigate the influence of male-immature affiliation on proxies of immature fitness in rhesus macaques. Here, we differentiate between ‘male care’ referring to affiliative interactions between adult males and unrelated youngsters and ‘paternal care’ referring to affiliation between sires and offspring. Using a second, independent data set, we further analyze whether the presence of sires during immaturity influences offspring lifetime reproductive success.

Rhesus macaques are a suitable species to study these questions for several reasons. First, females mate with several partners resulting in paternity confusion [36], which causes several males to potentially invest in a given infant due to their mating history with the infants’ mother [37]. Secondly, there is evidence that sires affiliate significantly more with their offspring than non-sires with unrelated infants, particularly during early infancy when infants are most vulnerable [21]. In addition, male rhesus macaques have been shown to provide care towards young independent of relatedness [21], which enables us to study both care provided by fathers and that provided by males more generally. Although male-infant interactions represent a relatively small proportion of the infant’s social network [21] they could effectively influence the fitness of infants if males provide protection or access to resources. On the other hand, females in this species are characterized as being restrictive mothers [38]. Although this behavior is likely to protect infants, it potentially reduces male access to infants, which could diminish possible effects on infant fitness caused by male-infant interactions.

The aim of our study was to test whether male-immature affiliation is beneficial for the immature fitness or proxies of fitness such as enhanced physical development [39,40]. We predicted three previously used proxies of fitness (body mass gain, body fat and testis size) to be positively affected by frequent affiliative interactions between adult males and young individuals because adult males can potentially enhance access to food by e.g., reducing feeding interruption [41] or allowing co-feeding [24]. Additionally, reduced amounts of stress received e.g., as a result of a secure male-infant relationship may also cause weight gain. Specifically, it has been shown that aggression results in an increase of stress level [42,43], whereas social bonding was found to decrease the stress level (ibid). Our chosen morphometric traits have previously been reported to influence infant/immature fitness. For example, body weight at three years of age influenced age of first reproduction in captive rhesus macaque females [44]. In male baboons, the onset of testicular enlargement indicates age of sexual maturation and thus the start of reproduction [25,45] with an earlier onset of sexual maturation subsequently translating into higher LRS (reviewed in [46]).

To assess the potential benefits of male-offspring affiliation for immature rhesus macaques we used two different approaches. On the one hand, we combined behavioral data on male-immature affiliation with morphometric measurements (proxies of fitness) of focal individuals taken at different stages of immaturity. For our behavioral data analyses, we chose five predictors of affiliative interactions between adult males and immature individuals, which potentially influence individual fitness. Based on previous findings suggesting that female sociality positively impacts infant fitness [40] *mean male-focal affiliation* was considered because a higher affiliation frequency with adult males can be expected to positively influence proxies of focal fitness. *Mean paternal affiliation* was considered, because sires were more likely to affiliate with their offspring than non-sires with unrelated infants [21]. *Focal rank* was included as a predictor, as immature rank might regulate the likelihood of male/paternal care, e.g., high-ranking immature individuals might be generally more attractive for caretakers. This can either be caused by males preferring high-ranking females as mates [47] (and associating with the female’s young, too) [48] or because high-ranking immature individuals are more likely to survive than low-ranking ones [49]. Furthermore, we explored *male-focal affiliation weighted by adult male rank*, because high-ranking affiliation partners, who often monopolize paternities [50,51] might have a stronger effect on our proxies of focal fitness than low-ranking ones [25], particularly when interacting more frequently. Finally, *affiliation distribution* was included for its impact on proxies of focal fitness as it is likely to increase social tolerance between partners and was shown to enhance food access in rhesus macaques [52].

Our second approach is based on long-term demographic and genetic data and was inspired by a previous study suggesting that co-residence of sire and offspring enhances the onset of sexual maturation in baboons [25]. Here, we extend the baboon study by investigating whether longer father-offspring co-residence during immaturity has a positive effect on offspring LRS. Assuming that co-residency enhances the likelihood of sharing spatial proximity, access to food or social interactions, we predict co-residency to influence offspring LRS, with the later shown to be a result of better physical condition [53–57].

Through both approaches, we test our overall hypothesis of whether male-immature affiliation or male presence during infant development is affecting infant fitness or proxies of infant fitness in rhesus macaques.

## Material and Methods

### Study species and population

Rhesus macaques live in multimale, multifemale groups with female philopatry [58] and male dispersal [59,60]. They are seasonal breeders [61] and both sexes mate with several partners [62].

The study was conducted on the free-ranging rhesus macaque population of Cayo Santiago (CS); a 15.2 ha island offshore Puerto Rico (USA). The population was founded in 1938, when 409 animals were captured in different locations in India [36], and is managed by the Caribbean Primate Research Center (CPRC). Although no individual has since been added to the population except via natural births, pedigree analyses reveal only rare cases of inbreeding (Widdig et al. unpublished). Females typically give birth to a single offspring [36], with an inter-birth interval of approximately one year. Thus, infants can be assigned to non-overlapping birth cohorts. However, infants from the same cohort may differ in age by up to six months.

Although the population is partially food provisioned (see below) and lacks predation, the field site is suitable for investigating both of our questions for several reasons. First, Cayo Santiago is home to several hundred habituated individuals in naturally formed groups. At the time of behavioral data collection, six social groups were present on the island, with group sizes ranging from approx. 80–300 individuals. Second, a unique genetic database is available for more than 4000 individuals spanning all social groups and several decades, which allows investigating both male and female LRS [63]. Third, demographic data including individual identity, date of birth and death, sex, group membership and matrilineal membership are available from census records continuously collected by the CPRC since 1956. CPRC census takers check group membership for at least two months following a potential migration event before noting a dispersal event. If a male remains consistently in the new group, the first day seen there is defined as the date of immigration. Finally, researchers are able to collect samples from and perform measurements on specific individuals during the annual trapping season.

The animals of the study population are fed once per day with a restricted amount of commercial high protein biscuits (0.23 kg/monkey/day), which are spread at three food supplies located in corrals. However, provisioning accounts for only 50% of the monkeys' daily food intake, with natural vegetation providing the remainder [64,65]. Nevertheless, high-ranking animals monopolize access to chow and tend to have higher weights than low-ranking animals (Langos pers. observation, [66]). Water is provided *ad libitum* at several drinking stations, where rainwater is collected in cisterns and distributed through pipelines.

In accordance with the CPRC Standard Operating Procedures for animal care, intervention and manipulation of individuals on CS is limited to (1) tetanus vaccinations and (2) severe medical situations. The latter occurs rarely, e.g., as a result of injuries caused by inter- and/or intragroup aggression between animals. Physical aggression from slapping up to biting is commonly used in rhesus macaques to establish or reinforce dominance hierarchy [62]. Animals are monitored daily and every time an individual shows signs of unhealthiness (e.g., disease, injuries) the status will be annotated and progression observed. During the entire study, none of the participating individuals from the focal animal behavioral data collection had to undergo any kind of medical treatment apart from the tetanus vaccination, which is administered to each individual at the age of one and two years during the two months of the annual trapping season conducted by the site management [67]. No animal had to be sacrificed.

During annual trapping, yearlings are marked with identification codes. Additionally, physiological samples of specific individuals may be collected for research purposes. In order to process individuals for blood sampling, body measurements or individual marking, live-trapped animals are transported to an enclosed area and anesthetized using an intramuscular injection of Hydrochloride Ketamine (10 mg/kg body weight). Blood samples are drawn via femoral venipuncture. Two blood samples with a maximum of 2ml of blood each is obtained.

### Behavioral data collection

#### (1) Data set

To investigate whether differences in male-immature association explained variation in proxies of immature fitness (body mass gain, body fat and testis size), we followed a total of 55 individuals born into the same age cohort (27 females, 28 males) from Oct 2004 to Aug 2008, starting immediately after birth. Focal subjects were members of troop R, which consisted of a total of 269 ± 23 (mean ± SD) animals across study years. At the end of the study period in Aug 2008, 28 focal subjects were still alive (15 females, 13 males) and had reached a mean age of 3.76 ± 0.06 years. Thirteen subjects died naturally during the study period due to injuries caused by physical aggression between individuals (see above) or for unknown reasons. Sixteen focal individuals were removed from the island by the CPRC as part of the colony management procedure and could therefore not be considered anymore after removal. Females in our study population undergo menarche at the age of approximately 2.5 years [68] and male capacity to reproduce starts at 3–3.5 years of age [36,69–71]. Although age at sexual maturation can be expected to vary between individuals [72,73], focal subjects most likely had reached sexual maturity by the end of the study. Since the behavioral analyses covered all developmental stages, we will refer to different life stages as follows: (i) infants (0–1 year of age), (ii) juveniles (from 1 year to sexual maturation (see above)), (iii) adolescents (from being sexually mature to reaching full adult body size; males approx. 3.5–8 yrs; females 2.5–4 yrs [74]), and (iv) adults. When using the term ‘immature individuals’ we refer to the time period between birth and sexual maturity.

We recognized all group members (>1yr) on an individual basis using individually distinctive features (e.g., faces, marks, body features) or the identification code. Some individuals younger than one year (without identification code) were marked with black dye if possible. Additionally, we confirmed the identity of younger subjects (<1yr) using the spatial proximity to the respective mother, assuming that young infants are more likely to be close to their mothers than to e.g. other adult females.

#### (2) Behavioral data

We observed focal subjects repeatedly from birth up to their 4^th^ year of age to cover the entire period until sexual maturation for both males and females. A total of 3543 observation hours was collected, resulting in 64.42 ± 37.33 (mean ± SD) hours per focal animal. We used a 20 min standard protocol (*focal animal sampling)* [75], with each subject being observed once per day at maximum. During each protocol, affiliative interactions between adult males and a given focal subject were continuously recorded. Affiliative interactions included socio-positive approaches i.e., no immediate agonistic interaction followed the approach (see [21] for details), as well as any friendly behavior like touch, hug, hold, groom etc. All interactions were recorded within the 2.0 m range of the focal animal and when the dyad would stay in close proximity for at least 5 sec (ibid). Focal observations were evenly distributed across the times of day and were conducted such that the number of protocols per subject and week was roughly equal. For more details on data collection (inter-observer reliability scores, software used, etc.) see [21]. Additionally, data on displacements, aggression or submission were collected *ad libitum* [75] to create a dominance hierarchy (see below). All observers were blind to the paternity status in the study group.

#### (3) Measurement of body condition and sexual maturation

To test for an influence of male/paternal care on the sexual maturation of focal subjects, we obtained morphological measurements on these animals during the annual trapping season lasting approx. 3 months from January to March.

*Body mass (BM)* of focal subjects was obtained at the ages of 0–4.5 months (infant period, range in days: 10–136), 12–17 months (juvenile period, range in days: 407–517) and 4–4.35 years (adolescence, range in days: 1502–1588) using a Taylor 60lb Utility Round Hanging Scale (IL, USA). Age ranges resulted from differences in birth date within the birth season as well as from differences in sampling date during the annual trapping season (see above). Individuals under anesthesia were carefully placed in a cotton sac and weighed. *Crown-rump length (CRL)* was measured from vertex to caudal tip of the ischial tuberosities [76] using a metal tape measure when the anesthetized individual was in left lateral recumbency. Testicles were measured in the fourth year of life using a digital sliding caliper (0–150mm; Profitexx; Hagen, Germany). In order to exclude the epididymis, the scrotal sac was stretched and the width and length of each testicle was measured [77]. *Testis volume (TV)* was calculated using the formula for a regular ellipsoid (4/3)*π*(L)*(W^2^), with W being the mean width and L the mean length of both testis (in mm), divided by the BM of the individual on the testis measuring day (following [78]). All measurements were taken three times at a given day and the mean value of each set was used in the analyses.

### Population demographic analyses

To test whether co-residence of father-offspring dyads during offspring immaturity had an impact upon the offspring’s LRS we used a second set of data. We extracted demographic and genetic data for 92 study subjects (30 females, 62 males) from the entire population who fitted the following criteria: (i) their sires were genetically determined, and (ii) subjects reached at least reproductive age and died naturally. These criteria ensured, that subjects had the chance to potentially reproduce and that we were able to control for the time of co-residency between offspring and sires. To avoid a potential cohort effect and to assure that subjects could potentially reach their maximum lifetime of approx. 25 years [71], the study subjects were extracted from 10 cohorts of which all individuals had already passed. LRS was defined as the number of produced offspring that survived the first year of life, i.e., the period of highest infant mortality [7] and time when individuals are genetically sampled in our study population.

### Genetic data—Parentage assignment

Maternity was available from long-term field observations (behavioral mother) and was genetically confirmed for all mother-focal dyads. Paternities of focal subjects were solved exclusively using the University of Leipzig genetic database [21]. All sampled males older than 1250 days (based on earliest age at reproduction [75]) and present on the island at least 200 days prior to an infant’s birth (mean ± SD gestation length is 166.5 ± 7.4 days [79]) were considered as potential sires for a given infant.

Paternity assignment of a given focal subject was based on a combination of exclusion and likelihood analysis with all mother-father-offspring trios genotyped on a minimum of 12 shared loci (mean ± SD = 16.35 ± 3.07 loci). Paternity was assigned to a specific offspring if the putative father had no mismatch with the mother-offspring dyad and the next candidate sire had two or more mismatches (strict rule, 53 trios) or 1 mismatch (relaxed rule, 2 trios) with the mother-offspring dyad. Assigned focal paternities were additionally supported at the 95% confidence level in favor of the male with the highest LOD score calculated by CERVUS 3.0 [80]. Further details on the parentage assignment of focal individuals are described in [21].

For investigating offspring LRS (second analysis), paternities of 92 individuals were assigned using the CPRC genetic database. This database currently consists of 4058 individuals typed at 27.8±0.4 (mean±SD) loci out of a panel of 28 markers (Widdig unpublished data, [63]). The mean number of alleles per locus was 8.6 ± 4.0, mean observed heterozygosity across loci was 0.692 ± 0.166, mean expected heterozygosity was 0.690 ± 0.165 and mean polymorphic information content was 0.652 ± 0.167 (calculations performed with CERVUS 3.0 [80]). There was no evidence of a null allele occurring on any of the loci and all loci were in Hardy-Weinberg equilibrium.

For 58 of the 92 individuals, a genetic sample of the mother was available and maternity from field observations could be genetically confirmed. We nevertheless assumed that the behavioral mothers of the remaining 34 individuals also were the genetic ones, because only ten out of 3695 mother-offspring dyads (0.27%) from the entire database could not be genetically confirmed. As a consequence, paternities were assigned using 58 mother-father-offspring trios and 34 father-offspring dyads. For 88 of the 92 individuals, the sire could be assigned according to the strict rule (57 parent-offspring trios and 31 father-offspring dyads). In two cases, paternity was solved in accordance with the relaxed rule (see above). For the remaining two individuals, the assigned sire had one mismatch with the offspring and the next candidate sire had at least 3 mismatches with the offspring. We accepted the latter two paternities due to the great exclusion power of the high number of loci. Moreover, we also confirmed all 92 paternities on the 95% level using CERVUS 3.0 [80].

### Ethics statement

Any handling procedure of individuals was approved and carried out in strict accordance to the rules and requirements of the CPRC and IACUC (protocol No. 4060105) and all efforts were made to minimize stress for the individuals.

### Data analysis

#### (1) Establishing dominance hierarchies

To account for changes in the dominance hierarchy over the study period we used the Elo rating method [81–83], which calculates dominance rank on a daily base. For this, we included the outcome of dyadic agonistic interaction among adult males recorded throughout the period of behavioral data collection [21]. The adult female hierarchy was also based on the outcome of dyadic agonistic interactions collected in 1997 (as used in [84] and developed by using the I&SI method [85]. Since 1997, the hierarchy has been updated continuously with agonistic data from long-term observations (Widdig, unpublished data). Given that the dominance relationships among sexually mature females were largely stable over time (as confirmed via *ad libitum* sampling over our entire study period), we assigned immature individuals an individual rank according to the rank of their mother, whereby offspring of the same female rank directly below their mother and inverse to the birth order (reviewed in [86,87]). To control for rank changes due to births and deaths of group members all ranks were calculated on a daily basis. Ranks within the male and female hierarchy (including focal subjects) were standardized separately per day to a range from 0 to 1 (lowest- to highest-ranking).

#### (2) Analyses of focal data

Initially, we attempted to run general linear models involving several predictors and interactions for each fitness trait. However, model diagnostics showed that the model assumptions were not fulfilled. Therefore, we were unable to run multi-variant analysis and conducted Spearman rank correlations between each fitness trait and each individual predictor. Whereas we previously aimed at using seven predictor variables, we decreased the set to the five best-grounded predictors for our study aim. In order to control for multiple testing we consequently conducted Fisher’s omnibus tests (FOT)[88] to assure correctness of P-values. The FOT controls whether any of the single P-value results truly is significant, or whether the significance potentially occured by chance. For this purpose all P-values for each set of predictor variables per focal sex were included in one chi-square distributed variable together with its degrees of freedom being twice the amount of P-values [89]. We computed each FOT in combination with a permutation procedure (running FOT 1000 times with randomized data) [90–92] to allow for the fact that P-values of subsequent analyses were not independent. P-values ≤ 0.05 were regarded as significant and those > 0.05–0.1 as a trend. We considered trends as well because dichotomising results due to P-values being significant or not can cause misleading conclusions [93]. Since sociality of immature males and females in our study species differs [94] and affiliation of adult males with male immature individuals is more likely than with female immature ones [21] we performed all focal-based analyses separately for male and female focal subjects.

*A*. *Body mass gain*. First we investigated the impact of male/paternal care during immaturity on the increase of focal body mass (A1). The increase in focal body mass was analyzed for two periods: period 1 between 3 months and 1.25 years and period 2 between 3 months and 4.25 years. For period 1, we used the estimated body mass of 47 focal animals (♂ = 24, ♀ = 23) at the ages of 90 days and 455 days (1.25 years), respectively, to calculate the estimated annual mass gain. For period 2, we used the estimated body mass of 28 focal individuals (♂ = 13, ♀ = 15) at the ages of 90 days to 4.25 years.

As individuals could vary in age by up to three months when weighed in a given year, we first normalized the body masses for each period to a standard age per period across focal subjects. For this purpose data for male and female focal individuals were pooled, as growth curves from immature rhesus macaques do not differ significantly in regards to sex [95]. The normalization was done in several steps. First, we fitted models with the body mass per period as response and focal age values (in days) as predictor. Ages were fitted as either linear, squared or logarithmic terms to find the growth curve for each period that fits best to our data. We used Akaike’s information criterion (AIC) [96] to choose the best fitting transformation, which was the squared transformation in all cases (the one with the lowest AIC). From the best fitting model, we took the residuals for each focal individual as a fixed deviation from the normal growth curve. We also took the fitted value for each standard age per period. This value was the assumed normal body mass at this age. Finally, we added the previously determined residual to this value for each focal subject, which produced the normalized body mass per focal animal at a specific age per period.

For reasons outlined above, we used five predictors in the behavioral analysis and tested these separately for male and female focal animals: (1) *Mean male-focal affiliation*, for which the mean affiliation frequencies of each focal individual with all males per day were calculated, (2) *mean paternal affiliation* representing the average affiliation frequencies between focal subject and sire per day, *(3) focal rank*, (4) *male-focal affiliation weighted by adult male rank*, which means that (i) high amounts of affiliation with high-ranking adult males lead to a large value, while (ii) low values result from low amounts of affiliation with low-ranking males and (iii) intermediate values can occur e.g., from few interaction with high-ranking males or many interaction with low-ranking males; and (5) *affiliation distribution* to evaluate whether affiliation per focal subject was equally distributed across male partners. For this purpose we computed the evenness [97,98] as a modification from the Shannon index [99,100]. Evenness was calculated per individual i.e., we divded the index of a specific individual by its maximum index possible. Values could range from 0 to 1, whereby values close to 1 indicate a uniform distribution of affiliation frequencies across partners, while 0 indicates that the distribution is skewed towards specific dyads.

Accordingly, we repeated the correlations for the weight gain between 0.25 and 4.25 years of age (A2) using the same approach as above.

*B*. *Quetelet Index*. As a second response measure, we used the QI (a proxy for body fat) from the subset of focal subjects still available when adolescent (having reached sexual maturity, but not yet fully adult). BM and CRL taken during the fourth year of life were used for calculating the *Quetelet Index (QI)*, a ponderal index used to estimate body fat in primates [76,101]. The QI was calculated using the following formula: QI = [BM in kg / (CRL in cm * CRL in cm)]*1000.

*C*. *Testicular volume*. In the next analysis we used the TV from male focal subjects at approximately four years of age (a proxy for the onset of sexual maturation) as the response to male/paternal care during focal immaturity.

#### (3) Population demographic analyses

*D*. *Life time reproductive success*. To test for the influence of father-offspring co-residence during offspring immaturity on offspring LRS, we used a GLMM with Poisson error structure and log-link function [89]. The response variable was the number of surviving offspring each male subject produced. In addition to father-offspring co-residence, we included focal sex and mother-offspring co-residence as variables in the model. Finally, we included the identity of mother and sire as well as the cohort and birth group as random effects together with random slopes of birth group and cohort within the fixed effect of father-offspring co-residence [102,103]. Prior to running the model we visually checked the distribution of the predictors and as a consequence log-transformed the variable mother-offspring co-residence. Moreover, we z-transformed values of mother-offspring and father-offspring co-residence, respectively, to a mean of zero and a standard deviation of one. We checked the model assumptions and found that overdispersion [104] was not an issue (dispersion parameter 0.405). Furthermore, we calculated Variance Inflation Factors (VIF; derived from a standard linear model excluding the random effects) [89], which indicated that collinearity was not an issue (largest VIF = 1.028) [105,106]. We assessed model stability by comparing the estimates derived from a model including all data points with those obtained from models with levels of random effects excluded one at a time, which indicated no influential cases to exist. To test whether the chosen predictor variables improve the fit of the model [103], we compared the fit of the full model with that of a null model (i.e., comprising only the random effects) using a likelihood ratio test [107].

The model was fitted in R version 2.15.3 [107] using the function “lmer” of the “lme4” package (version 0.999375 [108]). The likelihood ratio test was run by using the R function “anova” [108] from the R package “stats”. The VIFs were determined using the function “vif” of the R package “car” [109].

## Results

### Focal behavioral analyses

During the first period (within 1 year) males and immature individuals affiliated 1.78 ± 0.27 times per day with adult males (mean ± SD, range 1.31–2.54 times per day). The total number of males an immature individual interacted with was 23.36 ± 5.72 (mean ± SD, range 8–34).

Considering the entire immature period (period 2) males and immature individuals interacted still at the same level, with 1.79 ± 0.25 interaction per day (mean ± SD, range 1.35–2.57 times per day), while the total number of males an immature individual interacted with was 43.07 ± 7.83 (mean ± SD, range 29–57).

#### A1. Body mass gained within 1 year

Female infants gained 1.17 ± 0.17 kg (mean ± SD) during this one-year period, while male infants gained 1.22 ± 0.22 kg. Body mass gained by female focal individuals within the first year tended to correlate positively with focal rank and the amount of affiliation observed with higher-ranking adult males (Table 1). For male focal subjects, our results indicated a trend for a positive correlation between the affiliation distribution and increase of body mass. In other words, when affiliation of male focal individuals was equally distributed among adult male partners, body mass of male focal subjects tended to increase (Table 1).

**Table 1:** Results of Spearman rank correlations between predictors and proxies for immature fitness of focal subjects (analyses A-C; significant P-values shown in bold, trends resulting from FOT shown in *italic*).

Response variable	Predictor variable	rho ♂	P ♂	rho ♀	P ♀
Weight difference	Mean male-focal affiliation	-0.038	0.856	-0.076	0.733
0.25–1.25 yrs	Mean paternal affiliation	0.154	0.479	-0.098	0.648
N = 47 (24♂, 23♀)	Focal rank	-0.388	0.057	0.542	**0.008**
	Male-focal affiliation weighted by male rank	-0.214	0.317	0.445	**0.038**
	Affiliation distribution	0.49	**0.015**	-0.063	0.766
	Fisher’s Omnibus test	*0*.*062*		*0*.*057*	
Weight difference	Mean male-focal affiliation	-0.038	0.908	0.257	0.358
0.25–4.25 yrs	Mean paternal affiliation	-0.052	0.866	-0.492	0.066
N = 28 (13♂, 15♀)	Focal rank	0.137	0.657	0.2	0.468
	Male-focal affiliation weighted by male rank	0.335	0.263	0.014	0.965
	Affiliation distribution	-0.362	0.224	-0.057	0.839
	Fisher’s Omnibus test	0.652		0.466	
Quetelet Index	Mean male-focal affiliation	-0.231	0.446	0.271	0.333
N = 28 (13♂, 15♀)	Mean paternal affiliation	0.077	0.803	0.012	0.975
	Focal rank	0.181	0.555	-0.204	0.473
	Male-focal affiliation weighted by male rank	0.093	0.769	0.079	0.782
	Affiliation distribution	-0.274	0.36	0.118	0.677
	Fisher’s Omnibus test	0.761		0.533	
Testis volume	Mean male-focal affiliation	-0.049	0.888		
N = 13	Mean paternal affiliation	0.51	0.097		
	Focal rank	0.245	0.435		
	Male-focal affiliation weighted by male rank	0.091	0.781		
	Affiliation distribution	-0.007	0.991		
	Fisher’s Omnibus test	0.647			

#### A2. Body mass gained within 4 years

Female subjects gained 5.99 ± 0.62 kg (mean ± SD) during this 4-year period, while males gained 5.76 ± 0.51 kg. There was no significant correlation between any of the five predictors and the amount of mass gained over 4 years (Table 1).

#### B. Quetelet Index

Measurements of estimated BM and CRL of 28 individuals taken in the fourth year of focal animal life revealed a QI (proxy for fat) of 2.69 ± 0.20 kg/cm^2^ (mean ± SD) for females and 2.67 ± 0.20 kg/cm^2^ for males. There were no significant correlations between any of the chosen predictors and QI (Table 1).

#### C. Testicular volume

Testes of 13 males measured at the age of four years revealed a TV of 4.96 ± 1.77 cm^3^ (mean ± SD, range = 2.68–9.72), corrected by their estimated BM at 4.25 yrs. Correlations between the set of five predictors and TV were not significant (Table 1).

### Population demographic analyses

In our sample, sires and offspring were found in co-residence for 866.65 ± 572.43 days (mean ± SD, range 0–1461 days) whereas mothers and offspring were co-resident for 1391.97 ± 202.36 days (mean ± SD, range 459–1461 days).

#### D. Life time reproductive success

Males in this data set lived for 14.53 ± 5.13 years (mean ± SD, range 5.08–22.77 years) and sired 8.35 ± 9.12 offspring (range 0–48), while females lived for 16.65 ± 5.67 years (range 6.94–26.40 years) and had 10.27 ± 5.08 offspring (range 1–18).

**Fig 1:**
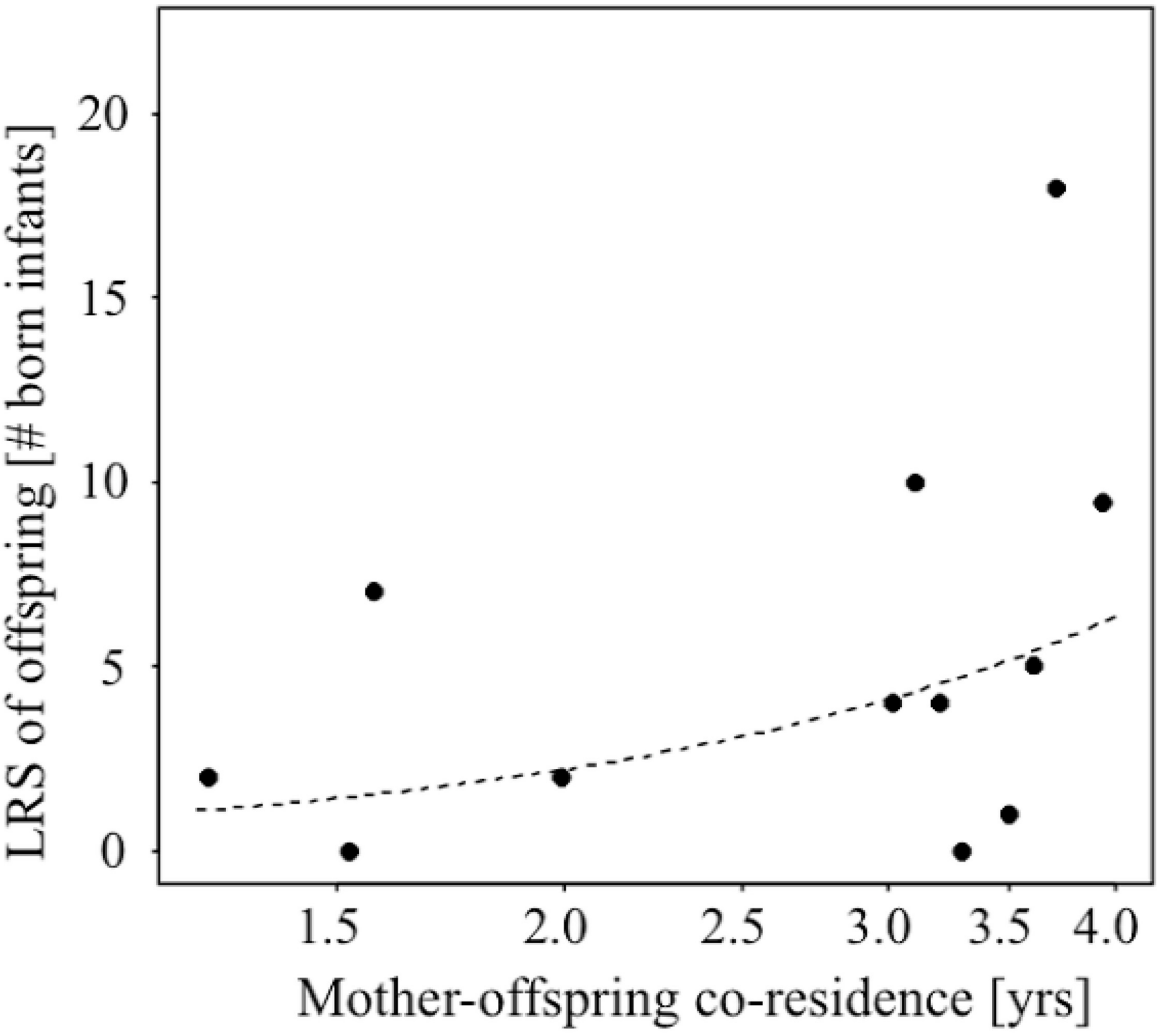
Influence of mother-offspring co-residence on LRS of offspring (line represents fitted model).

The comparison between the full and null model revealed a trend (likelihood ratio test, χ^2^ = 7.134, df = 3, P = 0.067), indicating that the predictors may have affected offspring LRS. Specifically, longer mother-offspring co-residence, but not father-offspring co-residence, led to significantly higher LRS in offspring (Table 2, Fig 1).

**Table 2:** Results of the GLMM examining effects of mother- and father-offspring co-residence and offspring sex on offspring lifetime reproductive success (population data, analysis D; z and p values not shown for intercept; significant value shown in bold).

Predictor variable	Estimate	SE	z	p
Intercept	1.973	0.164		
Sex	-0.251	0.158	-1.591	0.112
Co-residence with father	-0.056	0.089	-0.631	0.528
Co-residence with mother	0.367	0.129	2.247	**0.005**

## Discussion

Combining behavioral observations of male-immature affiliation with proxies of immature fitness, this study revealed a limited impact of male-immature affiliation on immature body mass gained during infancy for both males and females. In addition, our data confirmed the essential impact of mothers for offspring welfare, with mother-offspring, but not father-offspring, co-residence significantly impacted offspring’s LRS.

The results of our behavioural data analyses indicated that male-immature affiliation during infancy affected body mass gain of male and female focal individuals during their first year of life, but not during the entire immature period.

The proposed positive impact of male-immature sociality on proxies of fitness during infancy is in agreement with our prediction as well as with a number of primate studies that reported a positive impact of sociality upon individual fitness, e.g., in adult baboons (reviewed in [39]). The fact that a potential impact of male-immature affiliation on fitness proxies is restricted to infancy is generally in line with the evolutionary expectation that any kind of care directed to the young should be most beneficial to infants, as risk of mortality is highest during this early immature period [7,110]. The absence of the proposed influence over the entire immature period might be caused by the fact that male-immature relationships generally decline in rhesus macaques after reaching a peak at around two years of age [21]. Hence, our results are in line with the necessity for males to contribute to immature welfare during infancy when infants are most vulnerable [7], rather than during the entire period of immaturity.

In addition to the potential influence of male-immature affiliation on infant body mass gain, our results suggest a different impact on male and female focal subjects. Specifically, female infants seemed to gain body mass faster, when they experienced a higher frequency of affiliative interaction with high-ranking males. We limit the interpretation of our result to high-ranking males, since they interacted more often with females immature individuals than low-ranking males. Therefore, high values of the *male-focal affiliation weighted by male rank* were driven by interaction of female immature individuals with high-ranking males in our data set. In addition, female infants also seemed to gain body mass faster when their own rank was higher. For male focal individuals, body mass gain seemed to increase during their first year of life when the affiliative interactions were more equally distributed among their adult male partners. The suggested impact of rank on mass gain (female focal animal rank and adult male rank, the latter incorporated in a predictor with affiliation frequency) finds support in the despotic dominance hierarchy present in our study species [59,111]. In a variety of taxa, dominant individuals in despotic societies have priority of access to resources by means of foraging patterns (including level of feeding interruption) as well as quality of food (energy intake rates) [41,112–114]. Furthermore, the trend that evenly distributed affiliation among adult male partners positively influences body mass gain of male immature individuals is in agreement with previous studies reporting increased food access of immature individuals and their ability to reach higher quality food when associating persistently with adult males [52]. For example, improved access to resources is known to enhance body condition and age of maturation in a number of species [115–120]. Therefore, one may argue that male focal subjects with similar bonds even of medium strength to all their adult male partners may benefit from being tolerated at feeding sites by a larger number of males than male immatures who have very strong bonds with some males. Based on previous studies [39] one might generally expect that strong bonds with few particular males, implied by a skewed distribution in affiliation, should be more beneficial to immature individuals than weaker boonds with a larger number of males. However, in rhesus macaques, male aggression directed to immature individuals is rare [21], and costly male/paternal care behavior, such as the protection in agonistic encounters, is scarce [121]. Hence, stronger bonds to some males may not contribute more to immature welfare than bonds of medium strength to a larger number of males partners of medium bonding strength. More intense male-immature affiliation (by means of quantity) will probably not cause adult males to extend their care behavior from low cost care (affiliation) towards high cost care (protection) [27]. Another hypothesis for the potential benefit of a more evenly distributed male affiliation may be that the quantity of social interactions can affect the stress level of individuals in the way that higher sociality (more affiliation) is related to lower stress levels [122]. In turn, stress is related to reproduction and feeding opportunities as described in several studies [41,123,124].

The sex differences suggested in the adumbrated effects of male-immature affiliation to fitness proxies of focal subjects remain unclear to us. The lack of an influence of rank on body mass gain in male focal individuals may possibly be based on the fact that, male immature individuals benefit from interacting with adult males in general, independent from adult male dominance, because social patterns of male immature individuals develop differently than those of female immature ones, as shown in [94]. Moreover, male immatures were found to be less bonded with their mothers and maternal family compared to female immatures, probably as a result of enhanced maternal aggression during the first year of life (Kulik et al. unpublished). This may reflect the need of male immature individuals to socialize with other individuals than their maternal kin. In support of this hypothesis, affiliation between adult males and male immature individuals was more likely than between adult males and female immature ones [21]. Similar, males around the time of maturation were more likely to affiliate with their paternal kin than non-kin, as compared to females of the same age [125]. Overall, this suggests that the effect of adult male rank on male immature body condition may be absent or masked for focal male individuals whose affiliation was evenly distributed across all their adult male partners, including lower-ranking males present at the periphery of the group [126]. This is in contrast to female immature individuals, whose interactions with adult males may be biased towards high-ranking males located in the center of the social group. However, we did not test here whether female immature individuals interact preferentially with central males and male immature individuals with both central and peripheral ones, but we consider it a potentially interesting topic for future research.

One question that remains is why father-offspring affiliation, in specific, appears to have no impact on offspring fitness, although we previously found that father-offspring affiliations were more likely than affiliation between adult males and unrelated immature individuals in rhesus macaques [21]. Such effect might be masked, for example by maternal effects (see below). Future studies should investigate this further, especially in multimale, multifemale groups where father-offspring interaction are more frequently exchanged and potentially more costly than in rhesus macaques.

Apart from the evolutionary hypothesis that paternal care positively affects infant fitness [27] it has recently been suggested that males should provide care independent of their relatedness to the young [127]. Despite paternity uncertainty in rhesus macaques [128,129], males may care for young if the provision of care does not lead to a reduction in future reproductive success for males (low-cost care) [130–132] as has been suggested for our study species [21]. Moreover, male/paternal care may rather be driven by female mate choice and may therefore not necessarily lead to improvement of immature fitness. For instance, in cotton-top tamarins (*Saguinus oedipus*), adult males carrying young are more attractive to females as mates than those refusing to associate with young ones [133,134]. Also, adult male Barbary macaques caring for an infant prior to the breeding season had a higher chance of mating with the infant’s mother in the subsequent breeding season compared to males providing no care [28]. However, although rhesus macaque females exhibit some degree of mate choice [128,135], the ‘care-then-mate’ strategy is less likely in our study species, as unrelated males, in contrast to the prediction, do not bias their infant care when the mother is nearby. Instead, unrelated males provided more affiliation when the infants’ mother was absent [21]. This, however, would be a key to enhancing mating success with the infants’ mother [136]. Nevertheless, further studies incorporating mating success of caretakers are needed to investigate this question in more detail.

The results from the population demographic analysis revealed that longer mother-offspring co-residence during the entire immature period, but not father-offspring co-residence, promoted offspring LRS. This supports previous findings in mammals that demonstrated a major impact of the mother's presence on offspring welfare (reviewed in [137]). Our finding that father-offspring co-residency did not affect offspring LRS differs from studies in baboons that reported that father-offspring co-residency accelerates sexual maturation in offspring [25]. Specifically, daughters benefitted from co-residency with their fathers in general, while sons only did so when their fathers occupied a high dominance rank at offspring birth. However, as baboons typically gain dominance by contest [138,139], while rhesus macaques queue for dominance [111,140]), these species-specific patterns may lead to a different importance of male dominance for offspring. Alternatively, the effect of earlier sexual maturation found in baboons might not necessarily be transferred into lifetime reproductive advantages given that fathers generally spend only a proportion of time in the group of their offspring before further dispersal [141]. Unfortunately, we were not able to directly repeat the test of father-offspring co-residency on offspring sexual maturation with our available data. In the baboon study (using an aseasonal breeder) onset of testis enlargement was correlated with sire-offsring co-residence [25]. In an extended approach, we correlated male-immature affiliation with testis size of male focal subjects around puberty in rhesus macaques, but we found no significant effect. However, we were only able to measure testis volume during annual trapping occurring right before the start of the mating season. Previous studies suggested, that in seasonal breeders, such as rhesus macaques, the production of sperms is restricted to the mating season [142]. As the spermatogensis might not have been fully started when we collecting data on testis size (at maximum we collected these data one month before mating started; Langos, unpublished data), this might have affected our results. Hence, future studies would benefit from taking data of testis volume during the mating season.

The results of the population demographic analyses also showed that the variance in the number of offspring was larger in males than in females. Given the differential investment in their gametes, females consequently invest more in their gametes than males, while males contest over access to fertile females leading to a large variance in male than female fitness (Bateman’s principle) [16,143]. Our results are therefore consistent with theoretical assumptions and empirical evidence from previous studies [63,144–148].

Despite our comprehensive analysis, only one morphometric fitness proxy (body mass gain of focal individuals during infancy) seems to have been influenced by male-immature affiliation. The lack of further evidence in our study may have several reasons. Possibly, the influence on the fitness traits tested, although commonly used, is restricted to weight gain during infancy in our data set. Nevertheless, other fitness traits that we could not consider here may reveal other results. For example, age at menarche has been shown to impact an individual’s LRS (reviewed in [46]). Life history theory suggests that the early start of reproduction should maximize an individual’s LRS [149–151]. We suggest that immature individuals experiencing higher stress levels (causing reproductive dysfunction [57,152]) due to little or no care male/paternal received become sexually mature later than immature individuals receiving sufficient male/paternal associated with lower stress levels). In contrast, higher stress levels, due to e.g., fathers absence may also lead to accelerated maturation, as reported for humans [153]. Therefore, the onset of sexual maturation in relation to male/paternal care behavior might be worth considering in future studies.

Another possible explanation for the limited influence of male care on immature body condition, and the absence of paternal effects in specific, could be that in rhesus macaques, adult males, rather than immature individuals, benefit from male-immature affiliation. Hypothetically, male-immature relationships in rhesus macaques might lead to lower stress levels of adult males similar to grooming behavior, which reduced heart rate and glucocorticoid levels in non-human primates [53–56] and is known to positively impact fertility [57]. Other than that, male-immature association may also promote successful social integration of adult males [154]. Immature individuals might function as tools for males to facilitate immigration and form connections with females as shown in vervet monkeys (*Cercopithecus aethiops*). In this species, females bias their affiliative behavior towards certain males not only upon the males' dominance and tenure, but also upon the males’ friendly behavior towards her offspring [136]. As the amount of male-female sociality predicted male reproductive success in Cayo rhesus macaques [121], male-immature associations might thus increase the probability of reproduction for adult males.

The limited evidence of the proposed relationship between male-immature bonding and immature fitness in our study might in turn be caused by some characteristics of our study population. One may argue that fitness benefits may be weaker in food-provisioned populations such as the Cayo Santiago macaques [1], however, if such benefits are stable they should still be detectable. Furthermore, rank may have little or no effect on fitness on individuals of the CS population, as every individual might be expected to meet its nutritional requirements. However, our results contradict this argument, as they indicate that female focal rank affects body mass gain during infancy. This is most likely the case because individuals in our study population commonly fight over food due to pronounced intra- and intergroup competition [36]. Rank and fitness effects have also been described in captive groups of rhesus macaques, where provisioning and medical care is much more pronounced than in our study population [44,116]. Other than that, the likely increase in survival rates and/or smaller inter-birth interval in our study population due to provisioning and lack of predation probably results in a greater availability of maternal kin compared to wild populations. This may enable young individuals to compensate more efficiently for e.g., the loss of their mother, causing male care to be of potentially lower importance in the study population. Nevertheless, adult males should be able to provide better food access to immature individuals than female kin, as they outrank adult females and thus should be able to monopolize food items more successfully than females. This hypothesis is supported by our finding that more affiliation with higher-ranking males during infancy seemed to promote body mass gain in female focal animals.

Finally, maternal care characterized by a restrictive mothering style in our study species [38]. might mask fitness benefits derived from male/paternal care and could potentially explain the significant effects of mother-offspring, but not father-offspring, co-residence on offspring LRS in our current study. This could be due to the intense maternal care in primates being of predominant importance for immature welfare [1], which could also result in mothers limiting male access to infants. Comparative studies should investigate a potential link between maternal style and the extent of male/paternal care.

In conclusion, our results can be seen as another piece of a puzzle aiming to reveal the potential influence of adult males on offspring fitness, but also strengthen the need for long-term studies with a larger number of focal subjects that incorporate behavioral observations and fitness analyses at the same time [155]. Thereby, fitness benefits should be considered from the perspective of both partners involved in associations and fitness traits additional to those used here may need to be taken into account.

## List of Abbreviations

Abbreviations: Explanation
AIC: Akaike’s information criterion
BM: body mass
CPRC: Caribbean Primate Research Center
CRL: crown-rump length
FOT: Fisher’s omnibus test
GLMM: general linear mixed model
IACUC: Institutional Animal Care and Use Committee
L: length
LOD: logarithm of odds
LRS: lifetime reproductive success
QI: Quetelet index
SD: standard deviation
TV: testis volume
VIF: variance inflation factor
W: width
